# The role of action intentionality and effector in the subjective expansion of temporal duration after saccadic eye movements

**DOI:** 10.1038/s41598-020-73830-6

**Published:** 2020-10-09

**Authors:** David Melcher, Devpriya Kumar, Narayanan Srinivasan

**Affiliations:** 1 Department of Psychology and Cognitive Science, University of Trento, Trento, Italy; 2 Division of Science, New York University Abu Dhabi, Abu Dhabi, United Arab Emirates; 3 Centre of Behavioural and Cognitive Sciences, University of Allahabad, Allahabad, India; 4 Interdisciplinary Program in Cognitive Science, Indian Institute of Technology Kanpur, Kanpur, India; 5 Department of Humanities and Social Sciences, Indian Institute of Technology Kanpur, Kanpur, India

**Keywords:** Cognitive neuroscience, Human behaviour

## Abstract

Visual perception is based on periods of stable fixation separated by saccadic eye movements. Although naive perception seems stable (in space) and continuous (in time), laboratory studies have demonstrated that events presented around the time of saccades are misperceived spatially and temporally. Saccadic chronostasis, the “stopped clock illusion”, represents one such temporal distortion in which the movement of the clock hand after the saccade is perceived as lasting longer than usual. Multiple explanations for chronostasis have been proposed including action-backdating, temporal binding of the action towards the moment of its effect (“intentional binding”) and post-saccadic temporal dilation. The current study aimed to resolve this debate by using different types of action (keypress vs saccade) and varying the intentionality of the action. We measured both perceived onset of the motor action and perceived onset of an auditory tone presented at different delays after the keypress/saccade. The results showed intentional binding for the keypress action, with perceived motor onset shifted forwards in time and the time of the tone shifted backwards. Saccades resulted in the opposite pattern, showing temporal expansion rather than compression, especially with cued saccades. The temporal illusion was modulated by intentionality of the movement. Our findings suggest that saccadic chronostasis is not solely dependent on a backward shift in perceived saccade onset, but instead reflects a temporal dilation. This percept of an effectively “longer” period at the beginning of a new fixation may reflect the pattern of suppressed, and then enhanced, visual processing around the time of saccades.

## Introduction

A fundamental challenge for the brain is to synchronize sensory, cognitive and motor activity in order to effectively interact with the environment. A prime example of this challenge is the alignment of visual perception with saccadic eye movements. We typically make several saccadic eye movements per second, rapidly shifting gaze position. Each saccade creates a spatial discontinuity (change in retinal image) and a temporal gap of tens of milliseconds during which the eye is rapidly moving.

It has been known for some time that visual stimuli flashed around the time of saccades are often misperceived in spatial location and also in time^1,2^. The stopped clock illusion (“saccadic chronostasis”) represents one such distortion in temporal judgments around the time of saccades. When making a saccade to a clock with a moving second hand, people report the subjective impression that the time it takes for the clock hand to move, immediately after the gaze shift, is longer than usual. This subjective impression has been confirmed experimentally in the lab. In one study, a temporal counter was started at saccadic onset and the duration of the first interval after saccade onset was compared to a subsequent interval^3^. The post-saccadic interval had to be made shorter in order for it to be perceived as equal to the later standard interval, suggesting that the post-saccadic interval was expanded in time.

**Figure 1:**
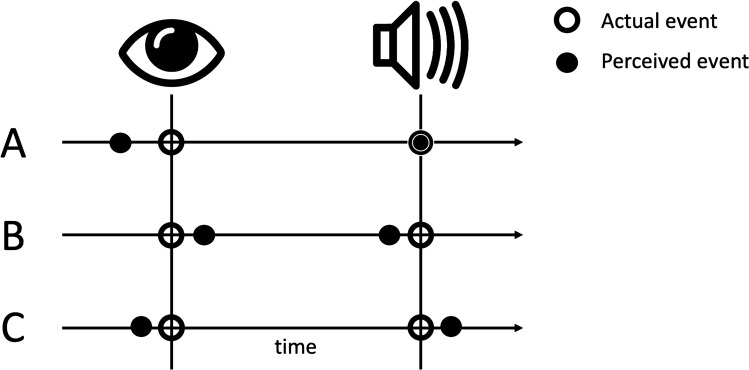
Three potential explanations that have been suggested for saccadic chronostasis and temporal illusions around the time of saccades. (A) According to the event backshift, or antedating, explanation, the post-saccadic period is judged as longer due to the timing of the saccade or the immediate post-saccadic input as being shifted backwards in time prior to the actual saccade onset. (B) According to intentional binding explanations, coming from studies of other action-consequence paradigms, the action and event are judged as closer together than actually occurred. Such compression effects have also been reported for saccades and saccadic chronostasis has been suggested as a compensatory effect to “undo” this binding. (C) Another group of explanations starts with the finding that the post-saccadic interval is judged as longer and suggests that enhancement of post-saccadic visual processing might underlie saccadic chronostasis. Image of the eye by Dave Gandy, used under Creative Commons Attribution-Share Alike 3.0 Unported license (https://creativecommons.org/licenses/by-sa/3.0/deed.en), https://fortawesome.github.com/Font-Awesome/.

There have been several competing explanations for saccadic chronostasis (Fig. 1) and this debate is complicated by the fact that the underlying neural mechanisms that allow for veridical time perception are a matter of debate^4–10^. In a recent review of the stopped clock illusion, Yarrow et al.^11^ identified three main potential explanations for chronostasis: action-backshift (or antedating) explanations, intentional binding leading to an event-shift, or temporal dilation of the post-saccadic time period due to, for example, increased attention or arousal. We will consider these three potential explanations below, as a motivation for the current study in which we aim to test these different predictions empirically.

### Action-backshift theories

One proposal has been that the illusion is based on a backward shift in the perceived onset of the saccade or its perceptual consequences (Fig. 1A), due to neural antedating or backdating^3,12^. In the initial Yarrow et al. study^3^, the difference in perceived duration of the post-saccadic interval was interpreted as evidence that the subjective timing of the immediate post-saccadic input (which was a counter that had actually started when saccade onset was detected) was shifted back in time. The perceived timing of the saccade was not directly tested, but it was inferred that the onset of the timer (which started at saccade onset), was extended backwards to around 50 ms prior to the start of the eye movement. Yarrow^13^ suggested that the illusion of temporal expansion of the period following the saccade could be because “our experience of time is effectively stitched up to take account of the saccade… to compensate for the period of degraded vision during the saccade.”

Backdating could be based on when the saccade was expected to occur, linked to a copy of the motor plan (efference copy, also known as corollary discharge^14^). The internal estimate of the saccade timing might be based on when the oculomotor plan is definitively decided but before the actual oculomotor muscles move, leading to an error of tens of milliseconds. Alternatively, the saccade might be actively suppressed from perceptual experience, with the missing time period during the saccade “filled in” backwards^3,11^.

One question about this type of explanation, however, is whether it is really necessary or efficient to “fill in” or “add time” every time we make a saccade. Such explanations seem to assume that there is an internal clock keeping track of the exact time of visual events in a continuous and metric fashion. If there is no such clock, as is widely argued^5–10,15^, then perhaps there is no need to continuously correct that clock every time we move our eyes.

A second issue with action-backdating theories is that “the interpretation of the experimental results requires an assumption about what can be seen during a saccade”, since “the duration of the comparison stimulus is often corrected before analysis to reflect the time it was foveated, rather than the time it was present on the screen”^11^. In other words, these studies assume that the actual starting time of the timer is not registered at all by the visual system. There is substantial evidence, however, that the visual system is in fact sensitive to high contrast information presented during the saccade^16–21^, calling into question whether some of the earlier research have overestimated the magnitude of chronostasis effect.

Further evidence for a backward shift in the perceived timing of the saccade comes from an earlier study in which participants were asked to judge the timing of a probe stimulus presented at the saccadic target before or after the saccade^22^. Participants rated the timing of the new fixation as earlier than it actually occurred, backdating the saccade to around 100 ms earlier than its actual onset. In a later study, observers reported the position of a second hand on a clock with respect to saccadic onset^23^. Here, the reported time that participants believed their eyes had landed was actually 40–60 ms prior to saccade offset. Although those studies did not investigate chronostasis, they provide evidence for the action-backdating explanation for the illusion.

### Intentional binding theories

An alternative explanation for chronostasis is related to a body of research showing that the sensory outcome of an action is perceived as shifted towards the time of the action^24,25^. Studies of “Intentional Binding” (IB) have shown that participants in lab experiments tend to underestimate the time between their action and a sensory outcome. This type of approach, which we will refer to as an “*event-shift*” account, is linked to the voluntariness of action^24^.

Interestingly, temporal expansion and compression have also been reported for other types of voluntary actions beyond saccades, including hand and arm movements^25–27^, leading to the idea that a volitional action in general, rather than a saccade specifically, might be the critical factor in the temporal illusion. At the same time, however, the direction of the temporal distortion reported has varied from study to study, sometimes showing evidence for dilation and sometimes compression. In terms of saccades, IB could be expected to shift the subjective judgment of the timing of the saccade and a post-saccadic event towards each other, leading to compression rather than an expansion of time (Fig. 1B).

Further evidence for compression in perceived timing or duration judgments comes from several peri-saccadic illusions^1^. When two bars are flashed around the time of saccades, for example, there is evidence for a compression of the perceived temporal interval between the flashed bars, as well as systematic errors in judging temporal order^28^.

An important distinction within the IB literature is between ‘freely-chosen’ actions and ‘cue-based’ actions, with strong IB found in the more intentional, self-paced actions conditions^24^. Experiments on IB have shown that when actions are believed to be self-initiated and causally linked to the outcome, the event is perceived to be shifted backward in time^29^. If chronostasis has a volitional basis, one would expect reduced effect for a cued saccade in comparison to a volitional one. Yarrow et al.^30^ compared a pro-saccade (where participants move gaze to a cue location) with anti-saccade condition (where participants make a saccade in direction opposite to a cued location), as well as comparing between self-timed, cued and express-saccade conditions. They failed to find any difference in subjective duration estimates and concluded that the mechanism underlying chronostasis is similar across different categories. Such an interpretation is compatible with the action backdating explanation but not with the IB explanation, which emphasizes the role of intention.

However, there are open questions regarding the role of intentionality in chronostasis. First, it is not clear whether a “self-timed” saccade always reflects a self-generated action rather than an action based on experimenters’ instruction. In Yarrow et al.’s study^30^, for example, the experimenter initiated each trial and participants executed the saccade only after a verbal “go” from the experimenter. These instructions leave the possibility that instead of being self-paced, the saccades were a form of cued pro-saccade with the cue being the verbal instruction from the experimenter. Secondly, Yarrow and colleagues considered *anti-saccade* trials as involving greater volition. It may be argued that although the anti-saccade trials require inhibition of an automated response, the participants might not link the saccade with their own causal agency. In intentional binding research, the voluntary nature of movement is typically manipulated across two dimensions: one, by allowing participants to freely choose the onset time of the action^29^ and second, by asking participants to choose among multiple actions^31^. The discrepancy in results from different studies raises the question of whether chronostasis (temporal dilation, action-backshift) and IB (temporal compression, event-shift) reflect a single underlying timing mechanism that might be influenced by intentionality and different measurement methodologies. Alternatively, these two experimental paradigms might measure two different aspects of the way that saccades (and other actions) interact with sensory processing and/or temporal cognition.

### Temporal dilation explanations

The third main explanation is that the post-saccadic time period involves a period of heightened neural processing, due to arousal or attention or other change in neural state^26^ that make the time period seem longer (Fig. 1C). Such explanations, however, are constrained by findings showing that chronostasis is similar for probe intervals ranging from 100 to 300 ms, suggesting that the effect should mainly occur in the time period of around 100 ms post saccade and then be carried over and added as a relatively constant effect independent of the interval in question. In other words, the whole time period after the saccade is not expanded in a multiplicative way, but rather a constant error is added onto the post-saccadic time period estimate^11^.

Another way of thinking of this third category of explanations is that saccadic chronostasis might not reflect an online, subjective dilation of time but rather a specific influence of saccades on the neural activity that is used as a proxy by participants in lab experiments to make temporal judgments (for a related proposal, see^5^). In other words, saccades may influence visual or cognitive processes related to the task (the way that participants in studies try to judge timing or duration), rather than altering perceptual experience per se. We typically make several saccades per second: does our subjective time actually expand and contract continuously? A more conservative explanation would be that we make an error when trying to make an explicit and retrospective temporal judgment when those judgments are made about events occurring around the time of saccades. If there is no single metric, internal clock tracking visual experience, as is now widely argued, then a temporal judgment task must be based on some other information^5–10,15^. Saccadic chronostasis may be an error in retrospection about the timing task, rather than an illusion in online perception^32^. Thus, an effect of the saccade on arousal, attention or neural activity more generally could influence the timing mechanism itself without influencing subjective perception.

An example of one such explanation, which focuses on timing mechanisms rather than online perception, is the well-known change in the sensitivity of visual neurons to sensory input around the time of saccades. These alternations effectively enhance post-saccadic neural processing^33–36^. Visual sensitivity decreases immediately before the saccade, while at the beginning of the new fixation the neurons in primary visual areas, and in other areas in the temporal processing stream, respond more vigorously to input than on average. This influences the temporal processing of visual input^37^ and could affect timing judgments in different ways.

One way that enhanced neural responses could lead to temporal expansion is due to the role of “magnitude” in temporal judgments (for review see^38,39^). According to some authors, different magnitudes in space or time (duration, length, and so on) are mapped onto the magnitude (amount of neural firing) of responses of particular sets of neurons. Since there is simply more response to the same stimulus at the onset of the new fixation, it would be judged as greater in magnitude. Some models of timing judgments for visual events build on evidence showing that V1 neurons respond quasi-linearly to stimuli as a function of their duration^40,41^. The increased magnitude of the response would lead to a shift in the perceived duration in higher-level cortical areas that turn early visual responses into temporal tuning curves that could be used in timing and duration tasks^42–44^.

Another way in which increased neural responses could lead to temporal dilation is that the estimate of duration is linked to the amount of processing that has occurred in that period^37^. Changes in neural responses at the beginning of a new fixation can be considered as more effective processing^45^. When more information is processed, this is correlated with a perceived expansion in time^37^. Conversely, temporal compression is correlated with less information processing^37^. Given evidence that information is processed more effectively at the beginning of the fixation, allowing more information to be encoded, then this would lead retrospective judgments of duration to overestimate the elapsed time.

We will refer to this group of explanations as a “post-saccadic enhancement” explanation of saccadic chronostasis, whether the enhancement is due to stronger V1 responses, arousal or attention (Fig. 1C). Such enhancement, compared to pre-saccadic suppression, could lead to both a perceived shift backwards in the estimate of saccade onset and also a forward shift in the estimate of an event occurring after saccade offset. This could account for the over-estimation of the duration of an event after the saccade as reported in the original Yarrow et al. paper^3^.

### The current study

The current study provides a test of the predictions from the three different explanations for chronostasis listed by Yarrow^11^: action-backdating (antedating), event-shift (intentional binding) and post-saccadic dilation in timing that leads to both a back-shift in saccade timing and a forward event shift (Fig. 1). One issue in comparing previous studies, and the inconsistent results, is that they have tended to use different behavioural measures. In order to make the results in the saccadic chronostasis phenomenon more directly comparable with those reported in the intentional binding literature, as well as previous studies estimating the timing of saccades^22,23^, we used the standard Libet’s clock task^46^ that has been used by a majority of the experiments investigating intentional binding. This allowed us to directly compare a typical IB paradigm to a saccadic chronostasis paradigm with the same measure.

**Figure 2:**
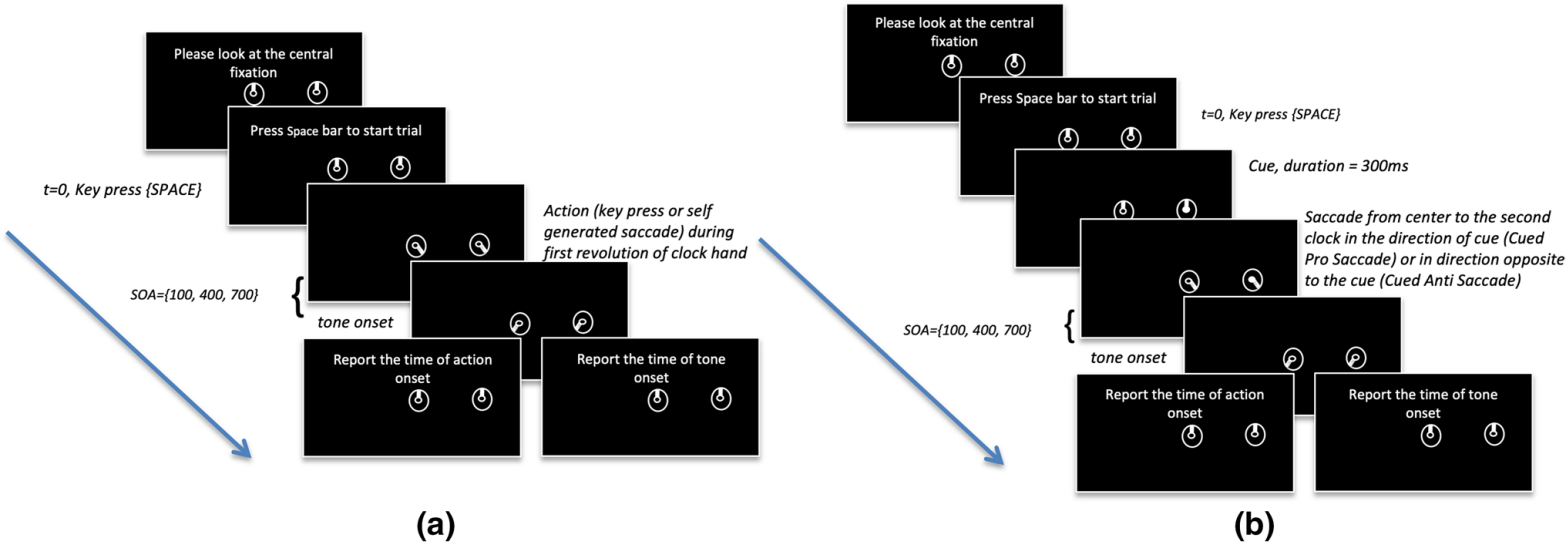
Order of main events in each experimental trial. (**a**) Trial structure for Voluntary conditions (**b**) Trial Structure for Cued Conditions. Each trial started with participants looking at central fixation and pressing the space bar key, at which point both clock hands started moving clockwise in synchrony at a fixed rate. Participants were asked to perform an action (either a keypress or a saccade), either in response to a cue (cued pro-saccade condition and cued anti-saccade conditions) or anytime during the first revolution of the clock-hand (self-generated keypress and self-generated saccade condition). The action was followed by a tone played after a varying SOA (100 ms, 400 ms, 700 ms). Participants were cued to report either the time of action onset or the time of tone onset, on separate trials.

On each trial, we asked participants to perform an action (saccade or button press) and then presented an auditory tone (Fig. 2). Participants were asked to report the position of the clock hand, either at the time of action onset or at the time of tone onset (varied in separate blocks). We also manipulated the voluntariness of actions. In each particular block, participants made a saccade either at their own will (self-generated saccade condition), in response to a cue towards that cue (cued pro-saccade condition), in response to a cue by saccading away from the cue (cued anti-saccade condition) or made a keypress instead of saccade (self-generated keypress condition). On each trial, the action was followed by an auditory tone presented at one of three temporal intervals (100 ms, 400 ms or 700 ms). In separate blocks of trials, the participants were asked to report either the perceived time at which they initiated a saccade or the time at which the auditory tone occurred (Fig. 2).

To compare the effects of chronostasis with that of intentional binding, we compared saccades for which the time of onset was volitionally chosen versus saccades for which the onset time was directly cued or instruction based. For ease of discussion we will call the former category of saccades as ‘self-generated saccade’ while the latter category as ‘cue-driven saccade’. It is important to note that this classification may not necessarily reflect how voluntary saccades are defined in the previous literature^3^. We are borrowing the classification from intentional binding literature, where it is used to distinguish between ‘freely-chosen’ actions and ‘cue-based’ actions^24^.

The current study allows us to better understand whether chronostasis shares a common mechanism with other temporal distortions related to action like intentional binding. Moreover, by testing the different motor effectors, tasks (action versus event timing) and volitional control (self-paced versus cued saccade onset), we were able to test the predictions of the different competing theories (Fig. 1). Testing action timing and event timing in the same study allowed us to characterize each of these separately, rather than inferring the timing mechanism based on a duration judgment. At the same time, we could infer perceived duration between the two events (action and event) and compare it to that found in previous studies. By measuring timing perception for an auditory beep, we also reduced the potential influence of temporal uncertainty, bias or attentional effects (such as visual priming, adaptation or prior entry effects) found with visual stimuli.

## Results

To investigate the patterns of temporal illusions for the four different motor tasks and three different auditory tone SOAs, we first examined tone timing and action timing judgments separately. We then considered the combined effects of the two measures by creating an estimate of the perceptual duration of the interval between saccade onset and auditory tone onset in order to test for potential intentional binding effects (compression of perceived duration between an action and a subsequent sensory event) or temporal dilation (expansion of perceive duration between action and event).

**Figure 3:**
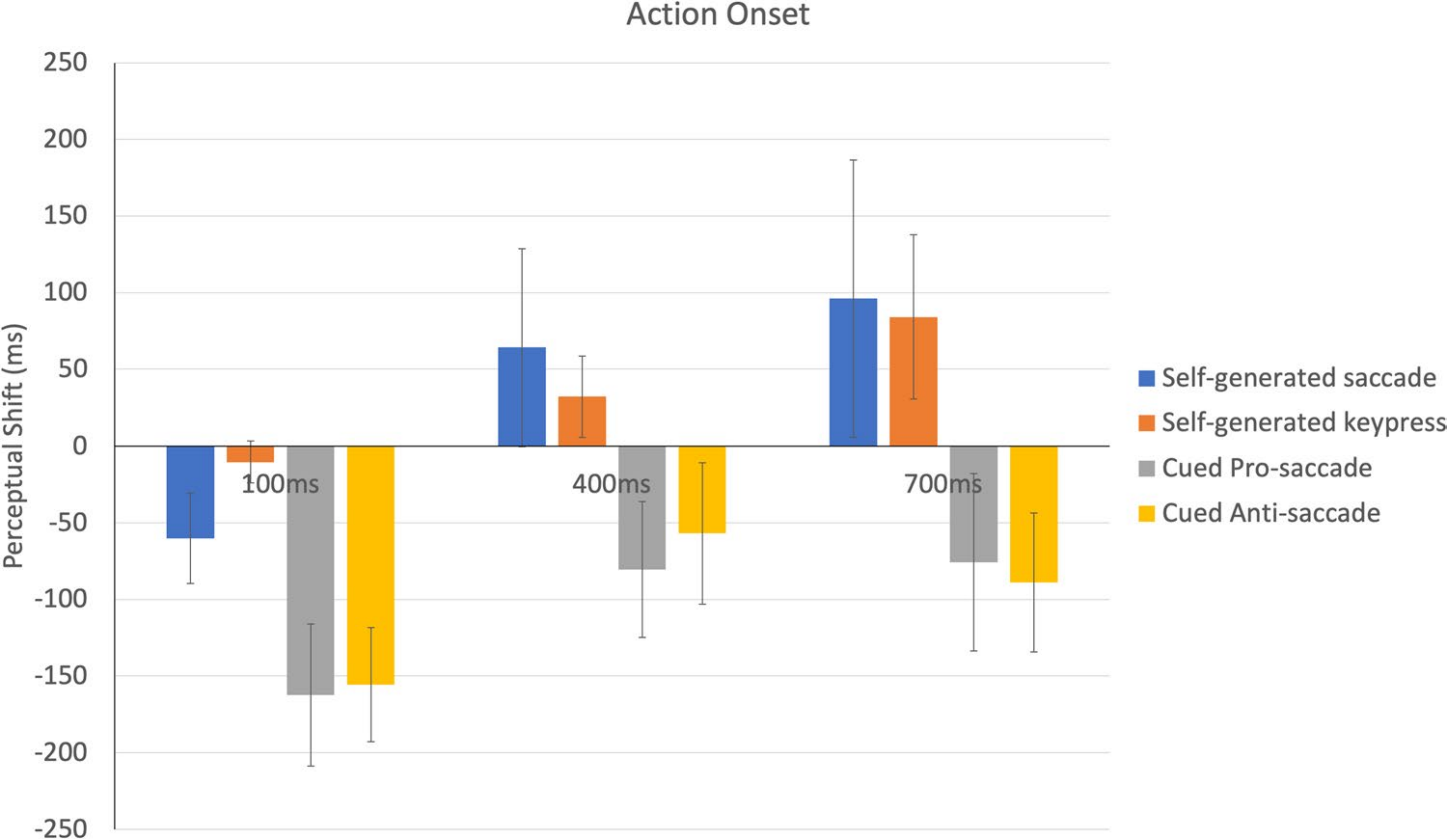
Shift in timing between veridical tone onset and reported tone onset, as a function of SOA and movement type condition. A negative value indicates a backward shift in time (reported as earlier than actually occurred) while a positive value indicates a forward shift towards later perceived onset time. Error bars indicate one standard error of the mean.

The perceptual shift in timing of the auditory tone onset, for each condition and SOA, is shown in Fig. 3. We performed a repeated measures ANOVA with action category and SOA as the within subject variables on the temporal shift for tone onsets. The main effect of action category was significant, *F*(3, 39) = 15.79, *p* < 0.001, η_p_^2^ = 0.55. Neither the effect of SOA, *F* (2, 26) = 0.98, *p* = 0.39, η_p_^2^ = 0.07, or the interaction between action category and SOA, *F*(1, 78) = 1.67, *p* = 0.13, η_p_^2^ = 0.114, was significant. Post hoc *t* tests (averaged across three levels of SOA) indicated a significant backward shift of tone onset for the self-generated keypress task compared to saccade conditions (self-generated keypress—self-generated saccade = − 87.15, *t*(39) = 7.6, *p* < 0.001, *d* = 2.43; self-generated keypress—cued pro-saccade = − 125.94 ms, *t*(39) = 11.06, *p* < 0.001 *d* = 3.54; self-generated keypress—cued anti-saccade = − 116.72 ms, *t*(39) = 10.2, *p* < 0.001, *d* = 3.26). For the keypress, this pattern of findings is consistent with previous reports of Intentional Binding (IB) for motor tasks^24,47^. In contrast, an opposite effect from IB was found in the saccade task, especially for longer SOAs.

**Figure 4:**
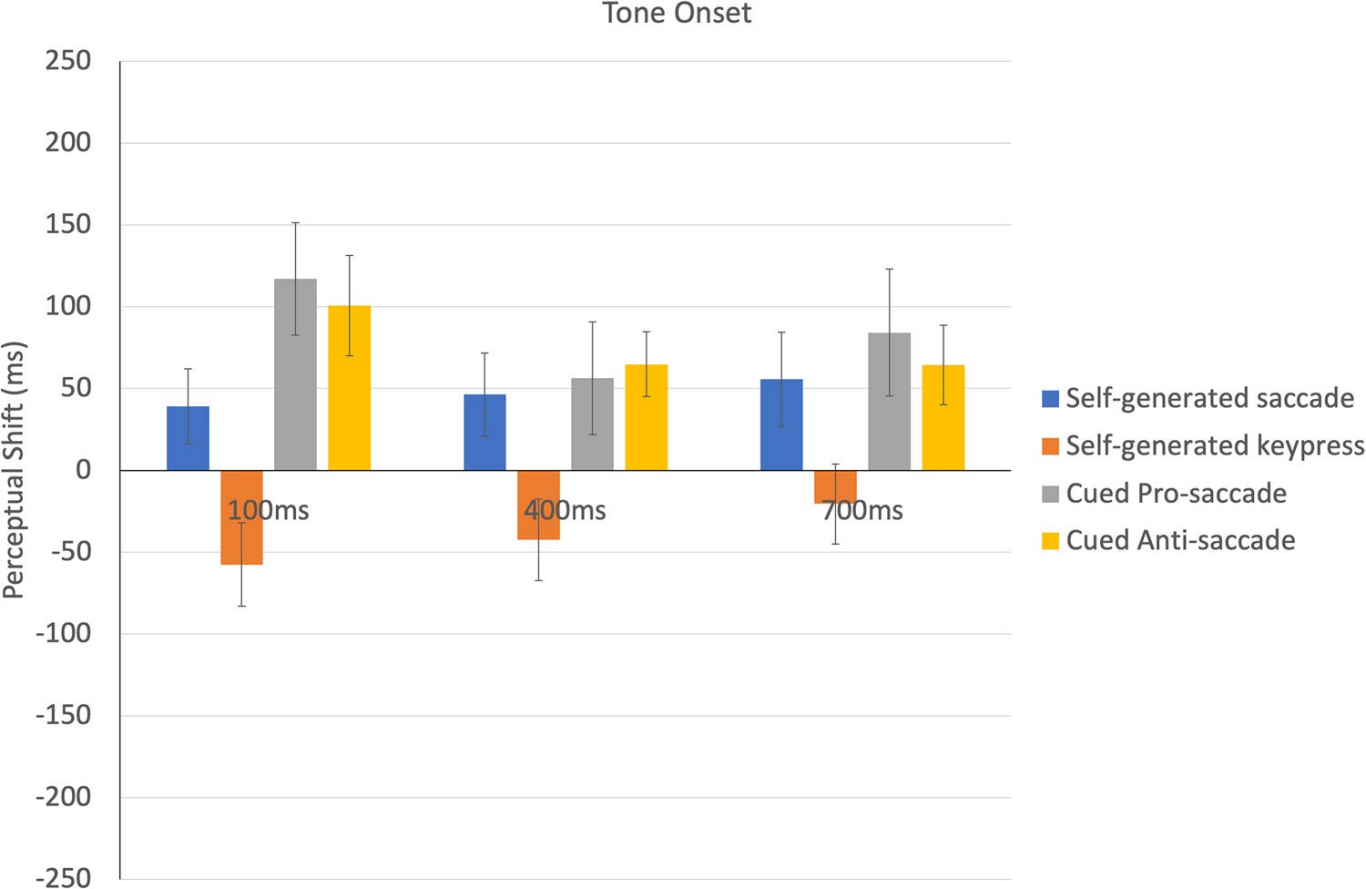
Shift in timing between veridical action onset and reported action onset, as a function of SOA and movement type condition. A negative value indicates a backward shift in time (reported as earlier than actually occurred) while a positive value indicates a forward shift towards later perceived onset time. Error bars indicate one standard error of the mean.

Next, we performed a similar repeated measures ANOVA for the reported timing for the onset of the motor action (see Fig. 4). The main effect of action category was again significant, *F*(3, 39) = 3.64, *p* < 0.05, η_p_^2^ = 0.22, but this time with both intentional actions (keypress and self-generated saccades) showing a forward perceptual shift compared to cued actions (keypress—pro-saccade = 141.57 ms, *t*(39) = 5.29, *p* < 0.01, *d* = 1.69; keypress—anti-saccade = 135.78 ms, *t*(39) = 5.08, *p* < 0.01, *d* = 1.62; self-generated saccade—cued pro-saccade = 39.68, *t*(39) = 5.22, *p* < 0.01, *d* = 1.67; and self-generated saccade—cued anti-saccade = 133.9, *t*(39) = 5.01, *p* < 0.01, *d* = 1.6). In addition, the main effect of SOA was significant showing a positive, forward shift in perceived event timing as a function of SOA, *F*(2, 26) = 6.39, *p* < 0.01, η_p_^2^ = 0.33. Post-hoc analysis suggests that for the 100 ms SOA, the action was perceived as occurring earlier compared to both 400 ms (100 ms—400 ms = − 86 ms, *t*(26) = 3.75, *p* < 0.05, *d* = 1.47) and 700 ms SOA (100 ms—400 ms = − 101 ms, *t*(26) = 4.36, *p* < 0.05, *d* = 1.7).

Thus, the overall pattern of results for perceived action timing was quite different from that found with tone onset timing, with the actions grouped by intentionality, rather than finger versus eye movement, and an effect of SOA with increasingly later judgments of the perceived action onset when the auditory tone was presented later in time. To further understand how intentionality influences the shift in time of action onset, paired t-tests between self-directed (averaged across keypress and intentional saccade) versus cued actions (averaged across cued anti- and cued-pro saccade) for the three SOAs were conducted. The difference was significant only for the 700 ms SOA, *t*(78) = 3.72, *p* < 0.05, *d* = 2.26. The main finding with the tone onset task was that the timing was underestimated (judged as earlier in time than veridical) in the keypress task but overestimated (judged as later in time than veridical) in all of the saccade conditions.

**Figure 5:**
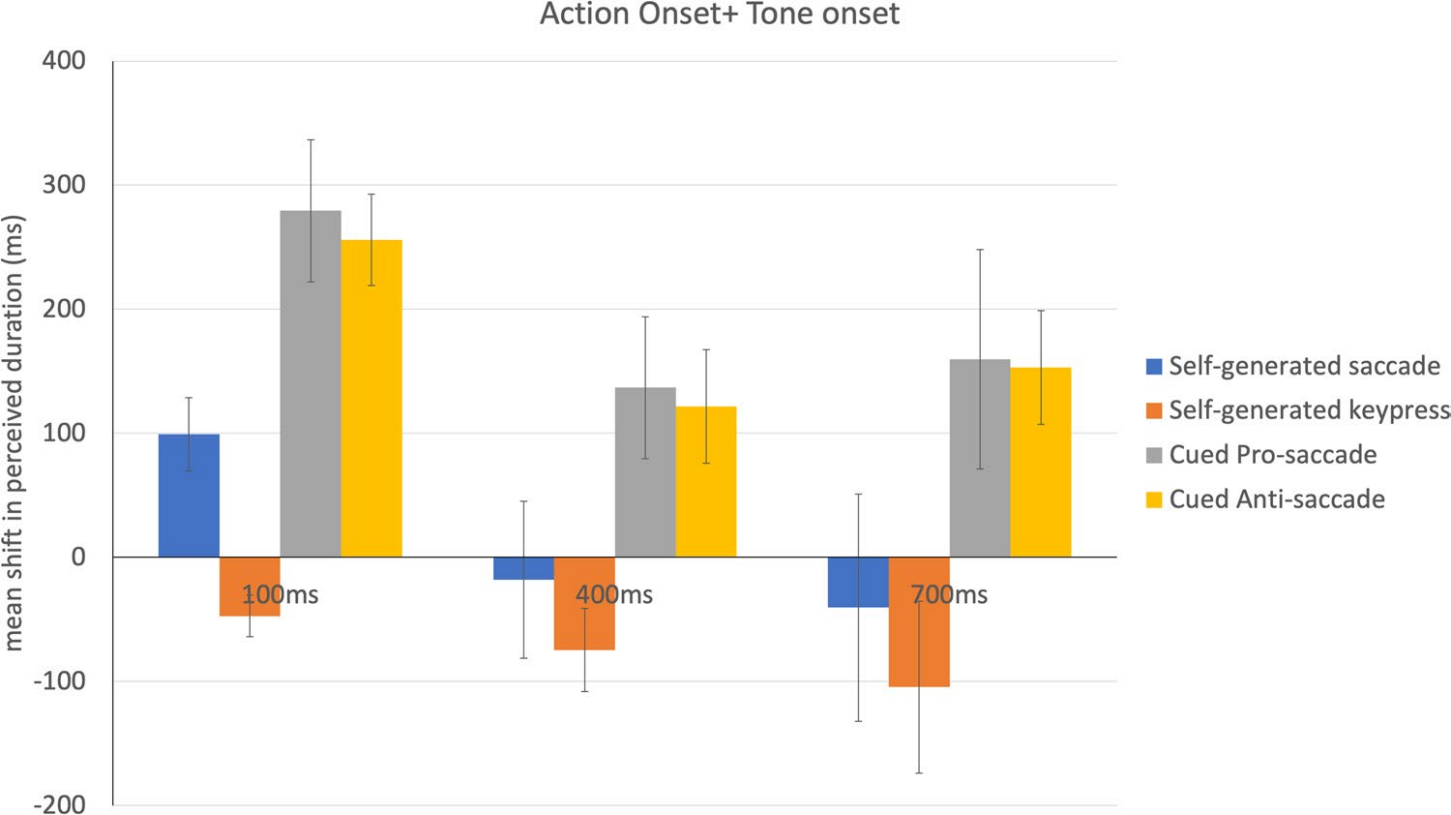
Shift in the estimated time interval between perceived action onset and perceived tone onset, for each motor task. A value near zero would reflect accurate timing. A negative value is consistent with temporal binding/compression while a positive value suggests subjective temporal expansion. Error bars indicate one standard error of the mean.

Based on this lack of independence between SOA and action judgments, it was then useful to consider temporal binding/expansion, measured as the degree to which the perceived timing of the action was shifted towards or away from the perceived time of the tone (Fig. 5). In this case, strong binding would mean that the two judgments are compressed together, which occurred only for the keypress condition. In sharp contrast, the two cued conditions (cued pro-saccade and cued anti-saccade) showed temporal expansion. This difference between tasks was confirmed by a significant main effect of action category, *F* (3, 39) = 8.97, *p* < 0.001, η_p_^2^ = 0.41. Post-hoc analysis (averaged across SOAs) showed that perceived durations for cued saccade trials was significantly less than that for pro-saccade, *t*(39) = 2.9, *p* < 0.05, *d* = 1.92, and anti-saccade conditions, *t*(39) = 2.37, *p* < 0.05, *d* = 1.76. Also, perceived duration for self-generated keypress condition was significantly less compared to pro-saccade, *t*(39) = 4.69, *p* < 0.01, *d* = 2.9, and anti-saccade conditions, *t*(39) = 4.96, *p* < 0.01, *d* = 2.7. The difference between self-directed saccade and self-directed keypress condition (*p* = 0.18) as well as cued anti-saccade and cued pro-saccade condition (*p* = 0.82) was not significant (Fig. 4). In addition, there was a main effect of SOA, *F* (2, 26) = 5.94, *p* < 0.01, η_p_^2^ = 0.31. Post-hoc analysis (averaged across action categories) suggests that binding was greater for 400 and 700 ms SOA compared to 100 ms SOA, *t*(26) = 4.10, *p* < 0.05, *d* = 1.6 and *t*(26) = 4.08, *p* < 0.05, *d* = 1.6 respectively. The difference between 400 and 700 ms was not significant (*p* = 0.58). The interaction between action category and SOA was not significant, *F*(6, 78) = 0.703, *p* = *0.6*5, η_p_^2^ = 0.05.

We also compared the saccadic latency for Cued Pro-Saccades (456 ms) and Cued Anti-Saccades (499 ms). A paired *t* test for log-transformed saccadic latency was significant, *t*(13) = 2.227, *p* = 0.044 (significant at α = 0.05). This 43 ms difference in saccadic latency is in line with earlier literature^48^. It is interesting to note that, despite the 43 ms difference in saccadic latency, there was no noticeable difference in the perceptual/timing effects found with Pro- and Anti-saccades.

Overall, the results replicated a classic IB effect only for the keypress condition, with the reported timing of the keypress misperceived as shifted later in time (towards the beep) and the auditory beep misperceived as shifted earlier in time (towards the keypress action). This IB was present for all SOAs but strongest for longer SOAs. The two cued saccade tasks (cued pro-saccade and cued anti-saccade) showed the opposite pattern. Participants dramatically overestimated the time between the saccade onset and the tone onset, in particular for the shortest SOA value.

## Discussion

The brain’s sense of time remains a lively topic of study and intense debate, defying a simple or single-mechanism explanation (for review^5–10,15^). Time perception includes estimates of temporal duration, either online or retrospective, as well as judgments of timing (when did the event occur with respect to a probe or clock) and temporal order. In the current study, we focused on the phenomenon of saccadic chronostasis, also known as the “stopped clock illusion”^3^. We measured judgments of the timing of an action (an eye movement or a finger keypress) and of a subsequent event (brief auditory tone) in order to characterize the mechanisms that might underlie the illusion. By including both a finger (keypress) and eye (saccade) movement, as well as manipulating the degree to which the movement was strongly volitional, we were able to test hypotheses from different theories of “the stopped clock illusion”, specifically, and of how the brain constructs (or reconstructs) timing judgements more generally.

In terms of the finger movements, we replicated previous findings of IB, with the timing of the movement and the tone shifted towards each other (compressed: see Fig. 5). This finding was important for three reasons. First, it showed that we were able to replicate a classic finding in perceived timing using our experimental set-up and measures. Second, it provided an example of an intentional, “self-generated” movement to compare to the self-generated saccadic eye movement (and contrast to the “cued” saccade). Third, by giving an example of temporal compression (action and event shifted towards each other), it allowed us to better compare it to any temporal dilation (action and event shifted away from each other) that we might find.

Interestingly, the exact pattern of the temporal compression for the self-generated keypress varied with the ISI of the tone. When the tone was presented immediately after the keypress (ISI = 100 ms), the strongest effect was in the backward shift of the tone towards the keypress, with little or no effect on the timing of the finger movement (Fig. 5). In contrast, at the longest ISI (700 ms), the largest effect was found on the action, which was reported as shifted forward almost 100 ms later than actually occurred, while the tone was back shifted less strongly. As described below, the fact that action and event timing judgments were not independent speaks to either a strong role for retrospective reconstruction of the events in the trial or, potentially, a temporal binding effect between action and tone limited to a 100 ms temporal window^15^.

Critically, none of the eye movement conditions resembled the pattern found with the finger keypress: we did not find IB with saccades (see Fig. 5). This finding is consistent with a recent failure to find IB in a study comparing a saccade condition with a more social gaze-following condition: the IB temporal compression was found only in the social gaze-following case^49^. In the current study, in both the cued conditions (pro-saccade and anti-saccade), we found a consistent combination of a backwards shift in event timing (saccades were judged to have occurred 80 ms or more before saccade onset) and forward shift in perceived timing of the auditory beep (to 50–100 ms later in time). Thus, we were able to replicate the classic saccadic chronostasis effect using the Libet clock task typically used in IB experiments. Based on these two temporal judgments, we can infer that there was an expansion of the post-saccadic interval.

Again, the timing of the auditory tone interacted with the way in which the saccadic chronostasis was manifested. In the self-generated saccade condition, when the beep was presented shortly after saccade onset (ISI = 100 ms), the pattern of results was somewhat like the cued saccade conditions with the timing of the saccade shifted back in time and both cued saccade conditions showing the strongest backwards shift, reaching around 150 ms prior to actual saccade onset. In the cued saccade conditions, this short ISI yielded the strongest temporal dilation, with the presentation of a 100 ms interval (between saccade and beep) judged as if it was over twice as long. For the longer intervals between saccade and beep (400 ms and 700 ms), the cued saccade conditions suggested an overestimation by about 100–150 ms (Fig. 4). This finding that the saccadic chronostasis effect was relatively constant in magnitude is consistent with previous reports^15^.

At the longer ISI durations, the self-generated saccades showed a unique pattern. There was a forward shift in the action timing estimate (like the keypress) but also a forward shift in the tone onset (like the cued saccade conditions). This suggests that there may have been some error in each of these judgments but the overall effect on estimated duration resulted in near veridical values (with both action and tone timing shifted forward in time by similar amounts).

The fact that the beep timing had any effect at all on the action onset timing suggests that the strategy used by participants was influenced by multiple factors and likely reconstructed retrospectively rather than based on online perception in real time. The timing of the beep influenced the perceived timing of both self-generated saccades and self-generated keypresses, even though it was irrelevant to action timing judgments. The finding of an interaction between the action onset timing judgment and the 100 ms tone onset condition, in particular, might also reflect an audio-visual binding interval of around 100 ms^15,50,51^.

Our findings are consistent with the existence of at least two separate mechanisms influencing timing judgments in this study. First, we found strong evidence for intentional binding (IB) in the self-generated finger keypress action and some intermediate evidence for IB in the self-generated saccade task. In the shortest beep timing (100 ms after the action), timing perception in the self-generated saccade effect was consistent with a combination (averaging) of the result in the keypress and the cued saccade tasks. Consistent with studies showing IB with a variety of different motor effectors^29^, our results provide evidence for a similar effect with saccades in the most voluntary “self-generated” condition, although this seemed to be somewhat cancelled by the second effect.

The second effect was time dilation, in the opposite direction of IB. This effect was strongest in the reactive, cued saccade conditions. This pattern is consistent with an over-representation of the time period immediately after the saccade. Interestingly, this occurred in all three saccade tasks, even in the self-generated saccade task (for the short tone ISI condition). Moreover, the shift in the tone timing was found for all saccade conditions and all values of the tone interval. Thus, it seems likely that the post-saccadic expansion in perceived duration found in saccadic chronostasis is not solely dependent on the backward shift in the saccade movement onset or the post-saccadic input, as has previously been suggested. In practice, the explanation of antedating as misperception of the “outcome” of the saccade (the initial moment that the post-saccadic stimulus is fixated, at the offset of the saccade) versus the timing of the saccade “action” (the saccade onset) is difficult to distinguish in the original chronostasis studies. Under either explanation, the key principle is that the duration of the post-saccadic interval is expanded. The current results suggest that this expansion can also generate a misperception in terms of a shift forward in events occurring during this post-saccadic interval.

Additional evidence for a general expansion of the post-saccadic interval, leading to both a backwards and a forward shift in timing of actions/events (depending on the measure), although interpreted differently by those authors, is the finding that the position of a moving target is perceived as shifted forward in space^52^. Interestingly, various control experiments in that study failed to find support for this forward spatial shift in terms of compensating for an illusory pause or backwards shift in the location during the saccade. Those authors then had participants directly report the perceived duration of the post-saccadic moving stimulus and found that the durations of the post-saccadic movement (which lasted between 250–750 ms) were overestimated by about 110 ms.

Our results also suggest that the intentionality of the saccade influenced the perceived duration between action and keypress. Yarrow et al.^30^ in their first experiment looked at pro-saccade and anti-saccade and found no difference in temporal distortions for the two types of saccade. In our experiment, the subjective duration for cued and anti-saccade conditions did not differ significantly (mean binding: − 200 ms), supporting their results. In their second experiment they did not find any difference between ‘Self-timed’, ‘Cued Pro’, and ‘Cued anti-saccade’ conditions, while we did find a difference between the self-timed and the cued conditions. This discrepancy might mean that the ‘self-timed’ saccade in Yarrow et al.^30^ may not reflect a free-willed action, given that participants in that study were given a verbal cue. Alternatively, the discrepant results may be due to the difference in the method used to investigate subject duration. The subjective duration for Yarrow and colleagues measured the distortion in time at the peri-saccadic and post saccadic intervals. In our study, the subjective duration was a compound measure of distortions at the time of action onset and at the time of tone onset.

In terms of intentionality, classical IB paradigms have typically varied intentionality along the “when” dimension (as appropriate, for example, for a finger keypress), which is what we have also manipulated in our study. However, the anti-saccade task often also varies the “where” aspect, making it unclear at the start of the trial what direction the saccade must go until after the “anti-cue” appears. To make the intentional and cued trials comparable, we made the cue only a temporal indicator of when to move, not where to move. This is a limit of the current study and so it would be interesting, in future work, to see whether varying intentionality in terms of “where” might influence perceived timing.

A second limitation of the study was in terms of its counterbalancing. Due to the large number of permutations, it was not possible to counterbalance all the conditions completely across participants. However, we ensured that no two participants received all the blocks in the same order.

Another limit with the current study is that there is no baseline condition of judging only tone onset (with no action) or action onset (without the tone). One potential problem with including an action baseline condition is that the type of actions differed across blocks (which is not the case in a classic IB study). This would have resulted in different baselines for different conditions, making any comparison between actions more complicated. Our conclusions are mainly based on comparisons of effects within action type (what is the shift in perceived action and beep timing, within each action type). Any general bias in timing would not account for the specific pattern of results found here. Nonetheless, it is important to interpret the implied interval between the event and the beep in relative, rather than absolute, terms.

When considering the role of intentionality, one interesting difference between the cued (saccade) and self-generated (saccade or keypress) conditions is the presence of an additional visual transient in the former. In addition to the smooth movement of the clocks, the 10 pixel in radius fixation point was filled in, as a temporal cue, in the cued-conditions. One possibility is that this small visual transient, given its importance in the task, might have attracted attention and then this cue could have been drawn towards the time of the action^53^. If the cue and the action were attracted towards each other in time, then this could have been expected to lead to a backward shift in the perceived timing of the cued pro-saccade and cued anti-saccade, and might explain some of the difference between cued and self-generated saccade conditions.

Overall, the current pattern of results is most consistent with a post-saccadic enhancement/dilation explanation (Fig. 1C) of saccadic chronostasis. As described in the “Introduction” section, our results would be predicted if saccades directly influence the mechanisms used by participants to make temporal judgments in this type of laboratory task. Numerous studies have shown that the same visual sensory input has more impact on neural responses at the beginning of a new fixation, either due to greater neural sensitivity, greater attention or arousal. Thus, a temporal judgment based on the magnitude of the neural response would then yield a longer estimate if, all else being equal, that stimulus is presented at the beginning of a new fixation. Given that many models of temporal perception and timing are based on using the magnitude of neural response as part of the input, this would allow for an explanation of saccadic chronostasis based simply on how people do such tasks and does not require an assumption that our online perception of time is repeatedly expanding and contracting as we look around the scene. This could provide a unified explanation for both saccadic compression^28^, during the interval before and during the saccade when visual input is processed less strongly, and then saccadic chronostasis for the beginning of the new fixation when visual neurons respond most vigorously to sensory input.

One potential reason for antedating the saccade action, or its consequences, is that processing of some visual input is suppressed during the saccade and so chronostasis may reflect the “filling in” of this gap^11^. Such “filling in” makes sense under the assumption that the stimulus presented on the screen at saccadic onset is completely suppressed from visual processing. Instead, there are many demonstrations that some visual processing continues during the saccade and, under certain conditions, we are able to see stimuli that were presented only during the saccadic eye movement^16–21^. In any case, we do also find that saccade onset is judged as earlier than it actually occurred, but this is combined with the tone being judged as later than it was presented. Thus, we find evidence that the judgment of an expanded interval after the saccade, found in the original studies, expands in both directions and not just backwards.

More generally, temporal distortions due to motor actions present a fascinating view of how our perceptual reality is constructed. The current findings provide a new perspective on saccadic chronostasis by showing both an influence of intentionality (consistent with IB theories) and a specific, saccade-related effect that was not found with the finger press. This saccade related change can be interpreted as a dilation or expansion of the estimated duration of the early parts of the new fixation. Whether the results are best interpreted in terms of saccade-related changes in neural sensitivity at the beginning of the new fixation, arousal, or in terms of action-backdating due to remapping and the efference copy, the “stopped clock illusion” suggests that the subjective and retrospective timing judgments in temporal cognition are influenced by a saccade. This finding is consistent with the existence of sensorimotor integration mechanisms^1–3,14^ that allow for perception to seem smooth and continuous across the dramatic shifts in the retinal image that occur several times per second in everyday life.

## Methods

### Participants

Based on a pilot study, for a power of 0.8 and α = 0.05, the computed sample size was 15. Fifteen volunteers (age range 20–25 years) from University of Trento with normal or corrected to normal vision participated in the experiment. All participants gave their informed consent to take part in the study. Data from one participant was not recorded correctly and was excluded from further analysis. The study design was approved by the Ethics Committee of the University of Trento and the experiment protocol for involving humans was in accordance with the guidelines of the Declaration of Helsinki.

### Stimuli and apparatus

Stimuli were presented on a 19″ LED display at a resolution 1024 × 768 with a standard PC connected to a Eyelink-1000 eye-tracker, which recorded the position of the participant’s right eye throughout the experiment. Behavioral responses were recorded using a standard keyboard.

Stimuli consisted of two “clocks”, one at the center of the screen and the other 10° away from the central fixation point, which was a hollow oval of size 10 pixels. In the cued movement blocks, a cue in form of a circle was presented 10° from the central fixation. The cue was a white circle with a radius of 10 pixels presented for a duration of 300 ms, which was used in cued blocks (pro-saccade and anti-saccade) to inform participants when they had to make a saccade. On each trial a 1000 hz tone was presented for a duration of 50 ms as an outcome of the participant’s action. The tone was generated using the “PsychPortAudio” function in PTB-3.

### Procedure

Participants were seated at a distance of 85 cm from the screen using a chin rest. The experiment consisted of two parts: a training block and the main experiment. In the training block, participants were asked to report the onset time of the tone using the clock. Every trial started with a centrally presented clock and a second clock either to the left or to the right of the central clock (counterbalanced across trials). Both these clocks were synchronized and had a random initial hand position for each trial. Participants were required to look at the centre of the central clock and press the space bar when ready. After the space bar was pressed, the clock hand started moving (2.56 s/per revolution). After an interval (100 ms, 400 ms or 700 ms) a tone was played while the clock hand was still moving. The clock hand kept moving after the tone offset and stopped at a random position after completing at least one revolution. The participant was then asked to report the position of the clock hand at the time of the tone, by moving the clock hand to its perceived position at the time of tone onset, using the left and right arrow keys (moving the clock hand clockwise and anticlockwise, respectively). Participants pressed the spacebar key twice to register their response.

In the training blocks, at the end of the trial participants were given feedback about their accuracy (perceived time of tone onset minus the actual time of tone onset). This training helped participants to perform better at the time estimation task. The training block consisted of 50 trials and took around 15 min to complete. No eye-tracking data was recorded during the training session.

The remaining experiment was divided into eight blocks (four action types × 2 blocks per action type) and conducted in two sessions (Fig. 2). Four experimental blocks were carried out in the first session and the other four blocks were carried out in a second session. Each session took around 90 min to complete. Before each block, a calibration of the eye-tracker was performed. Recalibration was done if at any time during the experiment the eye-tracker was unable to detect the position of fixation. Before each trial, the participant was asked to fixate at the center of the screen. When ready, they pressed the space bar key and then two clocks appeared on the screen, one at the center and the second clock located 10° visual angle to either the left or right side of the fixation cross (position counterbalanced within a block). The initial position of the clock hand was randomly chosen and was same for both clocks. The clock hands started moving at a fixed speed and took 2.56 s to complete one revolution. Participants had to perform the action (keypress or saccade) during the first revolution of the clock hand. In case a keypress or saccade was not detected within the duration, the trial was repeated. We manipulated the category of action across the blocks.

In the *keypress* condition, participants were instructed to make a keypress (up arrow key) at any time they wanted during the first revolution of the clock hand, while trying to keep the time of keypress random. In the *voluntary saccade* condition, participants were instructed to make a saccade to the center of the second clock at any time they feel like, during the first revolution of the clock, while keeping the time of initiation random. In the *reflexive pro-saccade* condition, a cue appeared at a random time during the first revolution of the clock hand. The cue was in the form of a white circle appearing at the center of the second clock. Participants were told to make a saccade, as soon as the cue appeared, in the direction of the saccade (any anticipatory saccades resulted in restarting the trial). In the *reflexive anti-saccade* condition, similar to the pro-saccade condition, a cue appeared at a random time during the first revolution of the clock hand but on the opposite side of the screen from the peripheral clock. Participants were instructed to make a saccade in the direction opposite to the cue, as soon as it appeared (any anticipatory saccades resulted in restarting the trial). Invalid trials, in which participants made a saccade to a non-target location or trials in which no saccade initiation was detected in the given time period, were discarded and repeated again.

For all of the conditions, after an SOA of 100, 400 or 700 ms post-saccade initiation an auditory tone of 1000 Hz was played for a duration of 50 ms. At the end of the trial, participants were asked to report either the time at which they initiated the saccade or the time at which the auditory tone was played.

The order of conditions was randomized across participants. All four action conditions were run in separate blocks, and the order of the blocks was randomized (without repetition) across participants. This meant that different participants started the experiment with different action blocks, randomly distributed. The response condition (action onset vs tone onset) was blocked and the order was randomized for each action condition. In other words, if participant 1 ran the keypress condition first, whether they made a judgment about timing of the action or the tone in that first block was randomized. After the experiment, it was confirmed that each participant had a different order of the blocks of trials (action condition × response type block). The SOA was randomized within blocks. Each condition consisted of 60 trials in which participants reported action onset and 60 trials in which participants reported tone onset, resulting in 120 trials per block and a total of 480 trials in the main experiment.

### Eye-movement analysis

Eye position measurements were classified into fixations and saccades by using a combination of velocity and acceleration threshold. An eye-movement was classified as a saccade when the velocity of saccade was greater than the velocity threshold and the rate of acceleration was greater than acceleration threshold. For the current study, based on pilot studies, the velocity threshold was kept at 150°/s and acceleration threshold was kept at 3000°/s^2^. Acceleration threshold was kept low due to potential issues in detecting anti-saccade movements which tend to have lower accelerations.

### Response analysis

We used 4 × 3 repeated measures ANOVAs to analyze the three dependent variables (perceived action onset, perceived tone onset and *binding interval* combining the shift in action onset and tone onset) as a function of *Action category* (self-generated keypress, self-generated saccade, Cued pro-saccade, Cued anti-saccade) and *SOA* between action and tone. For the effects that turned out to be significant, we conducted a post-hoc comparison between different levels of the factor, while averaging across other factors. The post-hoc comparisons were corrected for Type I error inflation using Tukeys’ method.

## Data Availability

Data is publicly available at the following link: https://osf.io/kxngt/?view_only=caf4cca2678e4fe2af290c3ba19a61f2
